# Acetate adaptation of *clostridia tyrobutyricum* for improved fermentation production of butyrate

**DOI:** 10.1186/2193-1801-2-47

**Published:** 2013-02-11

**Authors:** Adam M Jaros, Ulrika Rova, Kris A Berglund

**Affiliations:** 1 Luleå University of Technology, SE-971 87 Luleå, Sweden; 2 Michigan State University, 48824 East Lansing, MI USA

**Keywords:** *Clostridium tyrobutyricum*, Butyrate, Xylose fermentation, Hemicellulose utilization, Acetate inhibition

## Abstract

*Clostridium tyrobutyricum* ATCC 25755 is an acidogenic bacterium capable of utilizing xylose for the fermentation production of butyrate. Hot water extraction of hardwood lingocellulose is an efficient method of producing xylose where autohydrolysis of xylan is catalysed by acetate originating from acetyl groups present in hemicellulose. The presence of acetic acid in the hydrolysate might have a severe impact on the subsequent fermentations. In this study the fermentation kinetics of *C. tyrobutyricum* cultures after being classically adapted for growth at 26.3 g/L acetate equivalents were studied. Analysis of xylose batch fermentations found that even in the presence of high levels of acetate, acetate adapted strains had similar fermentation kinetics as the parental strain cultivated without acetate. The parental strain exposed to acetate at inhibitory conditions demonstrated a pronounced lag phase (over 100 hours) in growth and butyrate production as compared to the adapted strain (25 hour lag) or non-inhibited controls (0 lag).

Additional insight into the metabolic pathway of xylose consumption was gained by determining the specific activity of the acetate kinase (AK) enzyme in adapted versus control batches. AK activity was reduced by 63% in the presence of inhibitory levels of acetate, whether or not the culture had been adapted.

## Introduction

Butyric acid is approved by the Food and Drug Administration (US) as a flavor enhancer and several flavor esters used in the food industry are derived from butyric acid. There is a well established market for all-natural foods, where the components are not synthetically derived from petro-chemicals as well as a strong consumer bias against using genetically modified organisms (GMOs) in food production. Due to this, butyric acid fermented from biomass by wild type anaerobic bacteria can be developed as a saleable commodity.

Un-utilized hemicellulose streams from the pulp and paper industry can potentially, after hydrolysis, provide a low-cost source of xylose feedstock for organic acid fermentation. Hardwood xylan is extensively acetylated, i.e. up to seven acetyl groups per ten xylose units which facilitate xylose release by autohydrolysis (Teleman, et al. 2002). The resulting hemicellulose hydrolysate contains levels of acetate of up to 40 g/L acetic acid, inhibitory to microbial growth (Helmerius, et al. 2010). When used in fermentation media, the inhibitory acetate generates a long lag period before log phase growth and butyric acid production (Jaros, et al. 2012). Previous work has shown that addition of 17.6 g/L and 26.3 g/L acetate in the media generates a lag phase of 45 and 118 hours respectively while un-inhibited controls begin fermentation and subsequently production almost immediately upon inoculation (Jaros, et al. 2012).

 strains have been classically selected for increased tolerance to both butanol and ethanol which successfully lead to higher solvent yields and higher overall productivity (Lin and Blaschek Multiple *Clostridial*^1983^; Herrero and Gomez 1980). Due to the toxicity of these compounds, each step of the selection requires a short unchallenged incubation period, an exigency removed when challenging the organism with acetate.

For organic acid production, non-solventogenic *Clostridia* such a *C. tyrobutyricum* are used in fermentation processes where none of the typical toxic by-products such as butanol and ethanol are produced. *C. tyrobutyricum* cultures have been selectively adapted to tolerate the presence of inhibitory organic acids in order to increase acid product yields (Zhu and Yang 2003). Despite their success, these selections have been performed on immobilized *C. tyrobutyricum* cultures in fibrous-bed bioreactors requiring a 3 day cell growth period followed by a 36 to 48 hour cell immobilization period in order for a continuous feed fermentation to begin (Zhu and Yang 2003). Such a process allows for the eventual in-line adaptation of a *C. tyrobutyricum* culture to inhibitory acid products while simple adaptation techniques produce a tolerant culture ready to inoculate immediately into batch fermentation.

Through our work we have detected that *C. tyrobutyricum* demonstrates diauxic growth, the phenomena of a metabolic shift occurring in the middle of the growth cycle when the two carbon sources glucose and xylose are present (data not shown). The presence of a more utilizable carbon source, in this case, glucose, prevents activation of the metabolic machinery required for the cells to consume the secondary substrate, xylose. Fortunately, *C. tyrobutyricum* readily consumes xylose if the culture has been pre-conditioned to xylose metabolism and no other sugar sources are available.

Anaerobic, butyrate producing bacteria such as *Clostridia* metabolize glucose to pyruvate through the Embden-Meyerhof-Parnas (EMP) pathway and concomitantly generate acetate, butyrate, H_2_ and CO_2_ as major metabolic end-products (Zhang, et al. 2009). Xylose is specifically catabolised in the Hexose Monophosphate Pathway to pyruvate which is enzymatically co-oxidized with cellular coenzyme-A to acetyl coenzyme A (Zhu and Yang 2004; Madigan, et al. 2009). Acetyl-CoA is the branch-point node of the acetate and butyrate end-product pathways where the enzymes phosphotransacetylase (PTA) and acetate kinase (AK) are responsible for the metabolism of acetyl-CoA to acetate if the branch-point does not follow the butyrate pathway (2004). In attempts to force the carbon flux from the acetate to butyrate metabolic branch in *C. tyrobutyricum*, mutants have been developed with inactivation’s in the *pta* and *ack* genes coding for PTA and AK respectively (Zhu, et al. 2005; Liu, et al. 2006). Fermentations with the mutants yielded more butyric acid compared to wild type *C. tyrobutyricum*, but both mutant strains demonstrated significantly slower growth kinetics than wild type and in both cases resulted in higher final acetic acid concentrations with increased acid tolerance (Zhu, et al. 2005; Liu, et al. 2006). These results exhibit a common issue of genetic engineering in that GMO’s are typically less robust than wild type (slower growth) and the complexity of most metabolic pathways allows for the re-routing of inactivated processes due to homeostasis. The presence of 17.6 g/L to 26.3 g/L initial acetate in the media has the similar effect of lowering acetate production in xylose fermenting wild type cultures (Jaros, et al. 2012). This simple means of directing carbon flux towards butyric acid production is an added benefit of working with high acetate media and is especially important in light of evidence that such levels of acetate are present in potential xylose feedstock streams (Helmerius, et al. 2010; Jaros, et al. 2012).

*C. tyrobutyricum* batch fermentations under high acetate challenged conditions perform better with xylose as a carbon source than glucose. Fermentations with 26.3 g/L initial acetate generated 32.6 g/L butyric acid on xylose, while the comparable batch with glucose feed produced 22.3 g/L (Jaros, et al. 2012). Similar results were received with all initial acetate concentrations (0, 4.4, 8.8, 17.6 g/L). However batch fermentations utilizing high acetate (26.3 g/L initial acetate) xylose synthetic media resulted in an extended lag phase of 118 hours, lowering productivity (Jaros, et al. 2012). The extended lag phase generated by acetate is economically detrimental for batch fermentation of butyrate as it leads to a long period of reactor inactivity and potential exposure to microbial contamination. On the other hand, after lag phase the 26.3 g/L initial acetate challenged batch obtained a similar biomass concentration as the lower acetate and control batches and surpassed them in final butyrate yield (Jaros, et al. 2012). The focus of this work is to adapt a strain of *C. tyrobutyricum* to increased acetate tolerance, thus decreasing the extended lag phase while maintaining the acetate re-utilization metabolic mechanism to deliver increased yields of butyric acid. As hardwood derived hemicellulose hydrolysate feedstock gives rise to high levels of both xylose and acetate, a xylose consuming strain capable of overcoming the acetate induced lag and yet re-utilizing acetate to generate even more butyric acid would be of commercial value.

## Methods

### Microorganism and adaptation

A lyophilized stock culture of *C. tyrobutyricum* (ATCC 25755) was re-hydrated under sterile anaerobic conditions in Reinforced Clostridial Media (RCM; Difco). Once the culture entered log phase, when the optical density (OD) at 600 nm was approximately 2.0, transfers were made to glycerol stock vials (CRYOBANK™) and the culture was maintained at −70°C. *C. tyrobutyricum* was classically adapted to 26.3 g/L inhibitory acetate equivalents by serially passaging log phase cultures into serum bottles with RCM containing subsequently higher concentrations of sodium acetate (starting at 0 g/L then, 6 g/L, 12 g/L, 24 g/L and 36 g/L sequentially) at each passage. As the molar mass of sodium acetate is 82.03 g/mol, these concentrations correspond with 0 g/L, 4.4 g/L, 8.8 g/L, 17.6 g/L, and 26.3 g/L acetic acid equivalents respectively.

The adaptation was performed on two sets of *C. tyrobutyricum* cultures, each culture solely conditioned to consuming either xylose or glucose so that the actual batch fermentations could be performed without a lag phase due to an altered sugar source. The glucose conditioned culture was maintained with RCM from Difco with the appropriate additions of acetate equivalents in the form of sodium acetate. The xylose conditioned culture bottles also received the appropriate amount of acetate equivalent from a media consisting of: 10 g peptone (Fisher), 10 g beef extract (Teknova), 3 g yeast extract (Bacto), 5 g sodium chloride (J.T. Baker), 0.5g L-cysteine (Sigma-Aldrich), 3g sodium acetate anhydrous (J.T. Baker), 0.5 g agar (Bacto) and 900 mL distilled water. For the xylose feed, 5 g of xylose (Acros) in 10 mL distilled water, separately autoclaved at 121°C for 20 min was added to the culture media. Prior to autoclaving all serum bottles were sparged with nitrogen to maintain an anaerobic atmosphere. Each serum bottle contained a total volume of 100 mL RCM (initial pH 6.5) with 5 mL from the previous stage used to inoculate the next higher acetate stage. During adaptation, serum bottles were incubated at 36°C in an incubator-shaker (New Brunswick Scientific Innova 40) with shaking at 80 rpm.

The cultures required 24 hours to adapt and reach log phase growth before passaging to the next level of selection with the exception of the last transfer of the 17.6 g/L acetate adapted cultures to the final 26.3 g/L. Glucose conditioned cultures required 48 hours to reach log phase when challenged with 26.3 g/L acetic acid and xylose conditioned required 96 hours of incubation to reach log phase.

*C. tyrobutyricum* inoculum for each batch fermentation were pre-conditioned to the correct sugar substrate in the inoculation media prior the batch fermentation by anaerobically inoculating 50 mL Screw Cap Corning tubes containing 35 mL sterile glucose or xylose based RCM with 5 mL of the stock culture. The inoculated tubes were cultivated under anaerobic conditions at 36°C, 80 rpm, until log phase, approximately when OD_600_ had reached a value of 2.

### Fermentations

One liter batch fermentations were conducted in New Brunswick Bioflo 310 2.5 L working volume reactors under anaerobic conditions at 36°C. For each batch, 950 mL media of the following composition was used; 6 g/L yeast extract, 5 ppm FeSO_4_ 7 H_2_O, and 200 mL xylose or glucose at 300 g/L sterilized separately. Anaerobiosis was reached by sparging the vessel with nitrogen prior to inoculation. The batches were inoculated with 50 mL log phase *C. tyrobutyricum* cultures. The nitrogen sparging was maintained until logarithmic growth in the vessel was observed.

Agitation was kept at 250 rpm and in order to maintain the *C. tyrobutyricum* cultures in acidogenic production, pH 6.0 was sustained with 5 M NaOH throughout the fermentation. Sodium acetate (0 – 36 g/L) was added to the initial media prior inoculation for studies assessing acetate inhibition. Fermentations without acetate are referred to as controls. Samples (10 mL) were withdrawn at regular intervals for analytical measurements. Data presented in the tables and figures of this study are the results of single batch fermentations while an analysis involving duplicate and triplicate fermentations is given in the discussion where stated.

### Analytical methods

Organic acids and residual sugar were analyzed by HPLC (LC-20AT dual pump and 10A RI detector, Shimadzu) equipped with an ion exchange column (Aminex HPX-87H, 9 um, 7.8 mm x 300 mm, Bio-Rad) and a cation-H guard column (Micro-guard, 30 mm × 4.6 mm) using 50 mM sulfuric acid as a mobile phase. The flow rate of the mobile phase was maintained at 1 mL/min during analysis with 20 μL of sample injected into the system with an auto-injector (SIL-20AHT, Shimadzu) with the column and guard maintained at 65°C in a column oven (CT0-20A, Shimadzu). Prior to analyses, samples were centrifuged at 10 000 rpm for 5 min in a micro-centrifuge (Microfuge 18, Beckman Coulter). Data for each sample was acquired with Shimadzu EZ Start 7.4 SP1 chromatography software using standards for glucose, xylose, butyrate, acetate and lactate.

### Dry cellular weight determination

Cell growth was monitored during fermentation by measuring the optical density at 600 nm. The biomass from 40 mL cell suspension, removed in triplicate, was dried in an 80°C drier for 48 hours and the dry cell weight (DCW, g/L) determined. The optical densities were then converted to dry cell weight using the following equation: DCW = 0.38(OD_600_). This optical density to dry cellular weight conversion formula was determined for the specific organism and media used in this study.

### Specific Growth Rate (μ_net_)

DCW was used to determine the specific growth rate as described by Shuler *et al.* (Shuler and Kargi 2002). The DCW data points from the logarithmic growth phase were plotted on a semi-log graph to locate the period during that phase in which the culture experienced the fastest growth. These points were then used in the following equation: μ_net_ (1/h) = (ln(DCW_x_/DCW_0_))/(Time_x_-Time_0_), where DCW was measured in g/L and time in hours. DCW_x_ is the last point during the fasted logarithmic growth period and DCW_0_ is the first point. Time_x_ and Time_0_ are described similarly.

### Acetate kinase assay

Bacterial cells from xylose conditioned batches at log phase growth were chilled on ice and centrifuged at room temperature at 5,000 rpm for 5 min and washed in 25 mM Tris–HCl, pH 7.4 in order to remove acetate from the medium. After a second centrifugation the cell pellet was resuspended in 25 mM Tris–HCl, pH 7.4 and sonicated three intervals at 30 khz for 60 seconds, while on ice, to lyse the cell wall. The supernatant was used for acetate activity studies using a method (Rose 1955) where the conversion of acetate to acyl phosphates by acetate kinase is coupled to the formation of a ferric-hydroxamate complex detectable by UV–vis at 540 nm. In summary, the enzyme activity was measured at 29°C using UV/VIS spectroscopy where the absorbance of a 4 mL reaction mixture at 540 nm and the ferric-hydroxamate complex molar extinction coefficient of 0.169 mM^-1^ cm^-1^ was used to calculate the enzyme activity (Zhu and Yang 2003; Zhu, et al. 2005). Acetate kinase activity was standardized to the total protein content of each sample, determined separately by Bradford (Bio-rad protein assay) using bovine serum albumin. One unit of acetate kinase is defined as the amount of enzyme producing 1 μmol of hydroxamic acid per minute at 29°C and the specific activity calculated as units of activity/mg cellular protein. The results reported here are averages of enzyme assays run in triplicate.

## Results

### Fermentation kinetics

**Figure 1:**
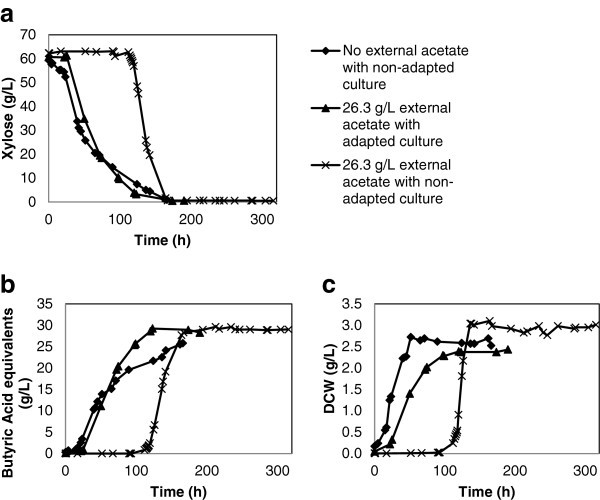
**Impact of acetate on xylose consumption, butyric acid production and biomass generation.**

The non-adapted (control) *C. tyrobutyricum* culture inoculated into xylose-minimal media begins sugar consumption almost immediately with butyric acid production beginning 15 hours later (Figure 1a and 1b). The same culture inoculated into xylose-minimal media containing 26.3 g/L acetate equivalents required over 100 hours to acclimate to the acetate despite both fermentations operating under the same conditions. The extended period of minimal metabolism and productivity is due to the acetate causing a delay in log phase cellular growth (Figure 1c). Once the *C. tyrobutyricum* culture had adapted to the 26.3 g/L acetate media the culture performed like the control, resulting in complete xylose utilisation and production of over 25 g/L of butyric acid and similar levels of cell mass.

The acetate adapted culture maintained tolerance to the 26.3 g/L acetate in the media and after a 22 hour lag in xylose consumption following inoculation, subsequently began producing butyric acid (Figure 1a and 1b). The acetate tolerant culture running under acetate inhibition conditions performed similar to the control fermentation in that the xylose was fully utilized in 175 hours from inoculation and produced 28 g/L butyric acid compared to the controls production of 25.8 g/L butyric acid. Despite the increased product yield, the net specific growth rate (μ_net_) of the acetate tolerant culture was reduced by 28.7% compared to the control. The specific growth rate of the control fermentation was 0.093 1/h while the acetate selected culture showed a log phase growth of 0.067 1/h (Table 1). This observation is not surprising as a similar yield increase corresponding with a growth rate reduction was seen in genetically modified *C. tyrobutyricum* where the *pta* gene had been deleted (Zhu, et al. 2005; Liu, et al. 2006).

**Table 1:** **Fermentation kinetics of*C. tyrobutyricum*cultures run in batch with or without selection for acetate tolerance and with or without acetate inhibition**

Sugar	Acetate	***C. tyrobutyricum***^***2***^	Lag time^3^	Complete utilization of carbon	Sugar cons^4^	Butyrate Yield^5^	Final concentration			Specific Growth Rate (μ_net_)^7^	Overall produc.^8^
	(g/L)		(h)	(h)	(g/L/h)	(mol/mol)	(g/L)			(1/h)	(g/L/h)
							Butyrate	Acetate	Bio- mass^6^		
Glc^1^	0	non-adapted	0	77	1.07	0.85	25.61	8.38	3.40	0.306	0.28
Glc	26.3	non-adapted	94	171	1.09	0.89	26.22	27.85	3.59	0.274	0.15
Glc	26.3	adapted	0	75	1.21	0.87	25.86	32.03	2.77	0.206	0.32
Xyl^1^	0	non-adapted	0	166	0.56	0.74	25.80	4.24	2.72	0.093	0.16
Xyl	26.3	non-adapted	102	167	1.22	0.79	29.00	27.76	3.04	0.121	0.12
Xyl	26.3	adapted	25	174	0.60	0.81	28.92	24.46	2.30	0.067	0.17

^1^ Glucose and xylose respectively.

^2^ Whether or not the inoculum had been selectively adapted to 26.3 g/L.

^3^ Calculated as time until sugar consumption started.

^4^ Calculated for the linear sugar consumption phase.

^5^ Yield was calculated as mol butyrate per mol glucose or xylose consumed during fermentation.

^6^ Calculated as DCW g/L.

^7^ As determined by the formula μnet (h^-1^) = (ln(DCW_x_/DCW_0_))/(Time_x_-Time_0_).

^8^ Overall productivity calculated from the start of the fermentation until the sugar source were completed.

**Figure 2:**
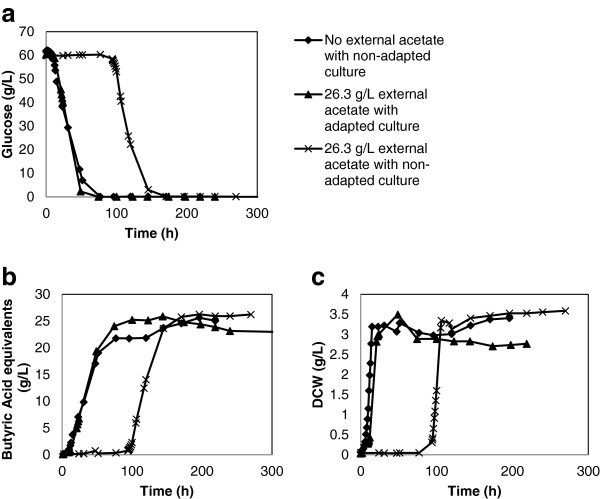
**Impact of acetate on glucose consumption, butyric acid production and biomass generation.**

The effectiveness of selective adaptation to generate an acetate tolerant *C. tyrobutyricum* culture is even more evident in glucose consuming fermentations. The adapted inoculum under 26.3 g/L acetate conditions experienced no lag in growth and tracked almost exactly with the uninhibited control in terms of glucose consumption and butyric acid production (Figure 2a and 2b). Unlike the xylose batches, the glucose consuming cultures (control, non-adapted-inhibited and adapted-inhibited) generated very similar levels of butyric acid between batches (25.61, 26.22 and 25.86 g/L respectively) (Table 1). Analogous to the xylose batches, the acetate inhibited non-adapted culture experienced approximately 94 hours of lag phase before beginning to consume glucose, produce butyric acid or generate DCW biomass (Figure 2a-c, Table 1). Acetate adaptation allows the culture to overcome inhibition caused by 26.3 g/L acetate and the 94 hours of lag phase. A net production of acetate occurred in the glucose consuming acetate adapted batch demonstrating the higher cellular energy made available from glucose consumption as compared to that of xylose. The xylose consuming acetate adapted batch activated the *Clostridial* acetate re-utilization pathway resulting in an overall consumption of acetate rather than production. This activation was likely necessitated by the lower amount of energy from xylose metabolism.

Similar to the xylose batches, the acetate tolerant culture consuming glucose also exhibited a 32.7% reduction in specific growth rate compared with the glucose control culture (Table 1). The glucose control batch demonstrated a 0.306 1/h specific growth rate and the adapted culture dropped to 0.206 1/h during acetate inhibition (26.3 g/L). The non-adapted culture under acetate inhibition (26.3 g/L) dropped to 0.274 1/h, only a 10.5% reduction compared to the glucose control batch.

The specific growth rates of glucose consuming batches were two to three times higher than those of the xylose consuming *C. tyrobutyricum* batches (Table 1). Lowered specific growth rates are a consequence of xylose consumption due to the lowered energetic value of xylose metabolism over glucose. With less free energy from sugar consumption, the xylose consuming batches have less energy to perform cellular maintenance and growth thus, in general have lower specific growth rates than glucose consuming batches.

The xylose consuming acetate-inhibited batches exhibited higher final yields of butyric acid than the control culture (Table 2). Both the acetate tolerant and non-adapted cultures yielded 0.48 g/g butyric acid from the initial 60 g/L xylose compared to the control cultures 0.43 g/g. Glucose consuming cultures demonstrated no significant change in butyric acid yield between the 3 batches (Table 2).

**Table 2:** **The effect of acetate inhibition on butyric acid yield in batch fermentations of*C. tyrobutyricum*with an initial 60 g/L glucose or xylose and run until completion**

	Butyric acid yield (g/g)		
Carbon source	No external acetate with non-adapted culture	26.3 g/L external acetate with non-adapted culture	26.3 g/L external acetate with adapted culture
Glucose	0.43	0.44	0.43
Xylose	0.43	0.48	0.48

The selection pressure during cultivation in 26.3 g/L acetate medium with xylose or glucose resulted in a strain with improved butyrate production while exposed to high acetate concentrations during fermentation. However, this phenotype was only preserved to some extent for the glucose fermenting acetate adapted strain. When this adapted strain, stored at −70°C, was used directly to inoculate a 26.3 g/L acetate challenged media, the lag phase was increased to 42 hours (results not shown), compared to 94 hours for the non-adapted strain. In contrast, there was a complete reversion of the acetate adapted strain during xylose fermentation using an inoculum from cryogenic storage. Further characterization of strain stability and the molecular mechanisms resulting in increased tolerance for acetate is needed to identify target enzyme pathways or individual genes important for the desired phenotype. The induced tolerance of *C. tyrobutyricum* enables one to use adaptation as a tool to identify alteration of the organism's own enzyme systems that can be targeted for further permanent genetic modification.

### Acetate kinase activity

**Figure 3:**
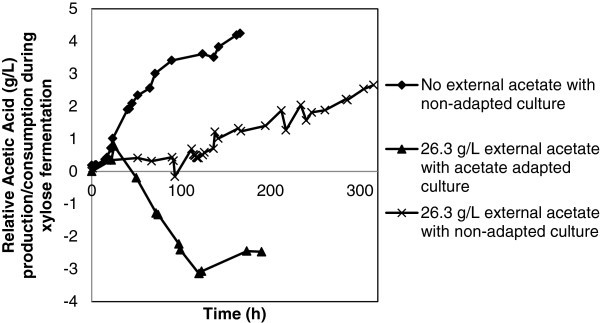
**Effect of acetate inhibition on relative acetic acid fermentation kinetics of*C. tyrobutyricum*xylose batches.**

The metabolic selectivity in *C. tyrobutyricum* is influenced by growth stage, with exponentially growing cultures producing both butyric and acetic acids, while slower stationary growth rates tend towards butyric acid (Michel-Savin, et al. 1990). As such, during log phase growth of each batch, culture samples were removed and analyzed for acetate kinase activity. Acetate kinase (AK) is the last enzyme on the metabolic arm converting acetyl-CoA to acetate, thus AK activity under particular fermentation conditions is related to acetate production (Liu, et al. 2006). Table 3 presents the specific activity in relation to cellular protein. The presence of inhibitory acetate (26.3 g/L) in the media reduced the AK activity to 3.15 U/mg in both the adapted and non-adapted cultures as the control culture exhibited 8.42 U/mg (Table 3). In both cases of acetate inhibition, whether the culture was acetate tolerant or not, the acetate kinase activity was reduced leading to the inhibition of metabolic acetate production (Figure 3, Table 1).

**Table 3:** **The impact of the presence of acetate on enzymatic Acetate Kinase activity in*C. tyrobutyricum*fermentations**

	No external acetate with non-adapted culture	26.3 g/L external acetate with non-adapted culture	26.3 g/L external acetate with adapted culture
Acetate Kinase activity (Units/mg cellular protein)	8.42	3.15	3.15

Results reported here are averages of enzymes assays run in triplicate as described in the methods.

The AK specific activity results correlate strongly to the production data in Figure 3, where the control culture with the highest AK activity also generated the most acetic acid equivalents, 4.24 g/L. The non-adapted batch with 26.3 g/L initial acetic acid equivalents and the lowered AK activity generated only an additional 2.65 g/L acetic acid by the time the xylose had been completely utilized (Figure 3). The selected batch run under the same initial acetic acid conditions performed with even higher carbon flux away from the acetate branch as acetate re-uptake mechanisms allowed the culture to consume 2.47 g/L of the initial acetate from the media (Figure 3).

## Discussion

Acetate tolerant *C. tyrobutyricum* cultures consuming xylose overcame the acetate induced lag growth phase four times faster than the comparable non-selected cultures under the same acetate inhibition conditions (26.3 g/L) (Figure 1a-c, Table 1). The selected culture also maintained lowered utilization of the acetate metabolic pathway under challenged conditions (Figure 3 and Table 3). The acetate producing metabolic pathway yields more ATP than the butyrate pathway, so an inhibition of acetate kinase (AK) or phosphotransacetylase (PTA) leads to increased carbon flux towards phosphotransbutyrylase (PTB) and butyrate kinase (BK) as the butyrate pathway must compensate for the energy loss (Zhu and Yang 2004; Michel-Savin, et al. 1990). Rather than lower energy consumption and less biomass generated, the acetate inhibited *C. tyrobutyricum* cultures generated a similar amount of biomass as the control by increasing butyrate production to overcome the energy inefficiency (Figure 1c, 2c). Similar to our results, *C. tyrobutyricum* fermentations with genetic inactivation of *pta* also had higher butyric yields and inactivated (or in our case, inhibited) acetate producers still developed similar levels of biomass as controls (Figure 1c, 2c) (Zhu, et al. 2005).

Both acetate kinase and phosphotransacetylase are more sensitive to product inhibition by butyrate than the enzymes responsible for the butyrate pathway, butyrate kinase and phosphotransbutyrylase (Zhu and Yang 2003). This natural inhibition is beneficial from an industrial standpoint as shortly after the culture enters the exponential growth phase *C. tyrobutyricum* stops co-producing both acid products and singularly forms butyrate (Michel-Savin, et al. 1990). The metabolic selectivity towards butyrate is further increased with the presence of acetate in the media as the acetate pre-adapted culture produced negligible quantities of acetic acid even during the beginning log phase stage (Figure 3).

Other than AK inhibition, another innate mechanism pushing the carbon flux of the *Clostridial* metabolism towards butyrate and away from acetate is the re-uptake of acetate from the media back into the usable acetyl-CoA pool by the CoA transferase enzyme (Michel-Savin, et al. 1990). This re-utilization mechanism of acetate provides no energy benefits to the cell but allows for the control of environmental acetate and utilizes protons in the acetate-to-butyrate conversion process (Michel-Savin, et al. 1990). Acetate re-uptake can be exploited under the conditions pertaining to a microbial inhibiting level of acetate present in the feed stream since the supposed contaminant in this case can potentially be used as a carbon source (Helmerius, et al. 2010; Jaros, et al. 2012). Some of the re-assimilated acetyl-CoA enters the butyrate pathway and thus this mechanism contributes to carbon efficiency (Canganella, et al. 2002). Acetate re-uptake occurred in the xylose consuming pre-adapted fermentation, not only is the final butyric concentration (28.92 g/L) higher than the control (25.8 g/L) but the initial acetate concentration decreases during the course of the study (Figure 1b and 3). Unfortunately, CoA transferase is also implicated in a redundant pathway leading to acetate generation directly from acetyl-CoA, so information concerning this enzymes specific activity may not provide useful information concerning the acetate re-uptake mechanism (Liu, et al. 2006).

The selective adaptation of acetate tolerant glucose consuming cultures completely eliminated the acetate induced lag phase in growth under inhibitory conditions (Figure 2a-c, Table 1). The higher energetic value of glucose consumption over that of xylose appears to allow acetate selected cultures consuming glucose to begin fermentation immediately even under 26.3 g/L acetate inhibition (Figure 2a). This is remarkable given that the non-selected glucose consuming batch still required a 94 hour lag-phase to overcome acetate inhibition, similar to the 102 hours seen in the xylose consuming non-selected culture under the same conditions (Table 1). The selective adaptation of *C. tyrobutyricum* for acetate tolerance is more effective for glucose consuming cultures than xylose consumers.

The energetic differences between xylose and glucose consumption appear to also affect the final butyric acid yields for 26.3 g/L acetate inhibited batches (data not shown). Duplicate fermentations of 60 g/L xylose produced an average of 27.16 g/L butyric acid with a standard deviation of (+/− 1.93) while duplicate fermentations of 60 g/L dextrose average 24.34 g/L butyric acid (+/− 0.99), a non-significant difference. Challenging the fermentations with 26.3 g/L acetic acid exacerbates the difference between carbon sources and leads to a significant increase in butyric acid yield or xylose consuming batches (data not shown). Given 26.3 g/L acetic acid inhibition, triplicate non-adapted batches consuming 60 g/L xylose generated an average of 30.45 g/L butyric acid (+/− 2.80) with duplicate batches of challenged glucose consumers producing only 25.20 g/L butyric acid (+/− 1.44).

The overall higher specific growth rates of glucose batches compared to the xylose batches is another result of the higher energetic value of glucose metabolism (Table 1). Due to this, the specific growth rates of the glucose batches are all two-to-three times faster than the corresponding xylose batches. As would be expected, acetate inhibition slows the specific growth rates in glucose batches but surprisingly, the non-adapted acetate inhibited xylose batch had a faster specific growth rate (0.121 1/h) than the control 0.093 1/h (Table 1). This can be explained by the long 102 hours of lag-phase that the non-adapted xylose batch had to adapt to the high level of acetate.

The overall butyric acid productivity of the non-adapted acetate inhibited xylose batch was only 0.12 g/L/h despite the faster specific growth rate. For industrial practices, the 102 hour lag-phase of the non-adapted xylose batch to start consumption is far too long a period of inactivity. The week of non-growth as the non-selected culture undergoes lag-phase would tie up fermentation capacity and potentially allow for contamination of the batch with other acetate tolerant microbes. The acetate adapted *C. tyrobutyricum* culture required only a 25 hour lag-phase until xylose consumption began, greatly reducing the time involved in complete batch fermentation.

The final yield of the selected acetate- challenged culture is 0.48 g/g (butyric acid/xylose), 0.05 g/g higher than control (0.43 g/g) (Table 2). This indicates the power of a simple selection method to adapt a culture which increases yield without the use of genetic modification. As one of the markets for bacterially fermented butyrate is as an all-natural food enhancer, a production process not utilizing genetically modified organisms might be a requirement.

## Conclusion

A simple selective adaptation for acetate tolerance generated a *C. tyrobutyricum* culture capable of reducing the acetate induced lag-phase by 75% for a xylose consuming fermentation and completely negated lag-phase in a glucose batch. Specific growth rates for acetate inhibited (26.3 g/L) batches of adapted cultures were reduced compared to non-inhibited control batches but despite this, the adapted cultures demonstrated greater overall butyric acid production than controls for either carbon source. Enzymatic data collected on acetate kinase demonstrated reduced activity in cultures fermenting xylose in the presence of acetate whether or not the culture had been selected for acetate tolerance. As selective adaption is a simpler technique to perform than genetic modification, the work here presents the potential for industrially producing all-natural butyric acid for consumer use.
